# Multivariate estimation of factor structures of complex traits using SNP-based genomic relationships

**DOI:** 10.1186/s12859-022-04835-3

**Published:** 2022-07-27

**Authors:** Ronald De Vlaming, Eric A. W. Slob, Patrick J. F. Groenen, Cornelius A. Rietveld

**Affiliations:** 1 Department of Economics, School of Business and Economics, Vrije Universiteit Amsterdam, Amsterdam, The Netherlands; 2 Medical Research Council Biostatistics Unit, School of Clinical Medicine, University of Cambridge, Cambridge, UK; 3 Department of Applied Economics, Erasmus School of Economics, Erasmus University Rotterdam, Rotterdam, The Netherlands; 4 Erasmus University Rotterdam Institute for Behavior and Biology, Erasmus School of Economics, Rotterdam, The Netherlands; 5 Econometric Institute, Erasmus School of Economics, Erasmus University Rotterdam, Rotterdam, The Netherlands

**Keywords:** SNP heritability, Genetic correlation, GREML, Genetic factor model, Genomic SEM

## Abstract

**Background:**

Heritability and genetic correlation can be estimated from genome-wide single-nucleotide polymorphism (SNP) data using various methods. We recently developed multivariate genomic-relatedness-based restricted maximum likelihood (MGREML) for statistically and computationally efficient estimation of SNP-based heritability ($h^2_{\text{SNP}}$) and genetic correlation ($\rho _G$) across many traits in large datasets. Here, we extend MGREML by allowing it to fit and perform tests on user-specified factor models, while preserving the low computational complexity.

**Results:**

Using simulations, we show that MGREML yields consistent estimates and valid inferences for such factor models at low computational cost (e.g., for data on 50 traits and 20,000 individuals, a saturated model involving 50 $h^2_{\text{SNP}}$’s, 1225 $\rho _G$’s, and 50 fixed effects is estimated and compared to a restricted model in less than one hour on a single notebook with two 2.7 GHz cores and 16 GB of RAM). Using repeated measures of height and body mass index from the US Health and Retirement Study, we illustrate the ability of MGREML to estimate a factor model and test whether it fits the data better than a nested model. The MGREML tool, the simulation code, and an extensive tutorial are freely available at https://github.com/devlaming/mgreml/.

**Conclusion:**

MGREML can now be used to estimate multivariate factor structures and perform inferences on such factor models at low computational cost. This new feature enables simple structural equation modeling using MGREML, allowing researchers to specify, estimate, and compare genetic factor models of their choosing using SNP data.

## Background

Narrow-sense heritability ($h^2$) quantifies the relative importance of additive genetic variance for a trait. Genetic correlation ($\rho _G$) reflects the shared genetic architecture between two traits. Genomic-relatedness-based restricted maximum likelihood (GREML) estimation [1–4] is widely used to estimate $h^2$ and $\rho _G$ using genome-wide single-nucleotide polymorphism (SNP) data for unrelated individuals [5]. As the heritability captured by SNPs provides a reasonable lower bound for $h^2$ [6, 7], the former is often referred to as SNP-based heritability and denoted by $h^2_{\text{SNP}}$ [8].

We recently developed MGREML, a computationally and statistically efficient approach for multivariate GREML [9]. Importantly, MGREML resolves inconsistencies when combining bivariate estimates into a multivariate $\rho _G$ matrix. By default, MGREML assumes the $\rho _G$ matrix is shaped by a so-called saturated model [10], which can fit any conceivable proper correlation matrix.

Here, we derive an extension of the statistical framework of MGREML (1) to estimate user-specified genetic and environmental factor models (e.g., a model with just one genetic factor for all traits) and (2) to test whether the given factor model fits the data better than a nested model (also user-specified).

Whereas Genomic SEM [11], another method to estimate genetic factor models, relies on preexisting summary statistics from large-scale genome-wide association studies (GWAS) for all traits of interest, MGREML uses individual-level data, giving users (1) more statistical power for a fixed sample size [9] and (2) more direct control over model specification and estimation (e.g., being able to control for an additional covariate in the MGREML analysis itself, rather than having to obtain a new set of GWAS results).

In short, we enable MGREML to estimate genetic and environmental factor structures using individual-level data, and to test whether a given factor structure fits the data better than a nested model. We validate this approach using simulations and an empirical application.

## Implementation

### Model

Consider a set of *N* unrelated individuals for whom we observe *T* traits, *k* covariates, and *M* SNPs. Let ${\textbf{X}}$ denote the $N \times k$ matrix of covariates, ${\textbf{G}}$ the $N \times M$ matrix of standardized SNPs, and ${\textbf{Y}}$ the $N \times T$ matrix of traits, for which column *t* corresponds to Trait *t* and is denoted by ${\textbf{y}}_t$. Furthermore, let ${\textbf{S}}_t$ denote a binary $K_t \times k$ matrix indicating which of the *k* covariates in ${\textbf{X}}$ apply to Trait *t*. Now, the matrix of covariates for Trait *t* can be defined as ${\textbf{X}}_t = {\textbf{X}} {{\textbf{S}}}^{\top }_t$.

When applying univariate GREML as implemented in GCTA [1] to Trait *t*, the following linear mixed model (LMM) is estimated using restricted maximum likelihood (REML) [12]:

$$\begin{aligned} {\textbf{y}}_t \sim {\mathcal {N}} \left( {\textbf{X}}_t \varvec{\beta }_t, {\textbf{A}} \sigma _{G_{tt}} + {\textbf{I}} \sigma _{E_{tt}} \right) . \end{aligned}$$

In this model, $\varvec{\beta }_t$ is the $K_t \times 1$ vector of fixed effects of the covariates that apply to Trait *t* and the $N \times N$ matrix ${\textbf{A}}$ is the so-called genomic-relatedness matrix (GRM) reflecting the subtle genetic similarities between unrelated individuals.

Typically, the GRM is calculated as ${\textbf{A}} = M^{-1} {\textbf{G}}{{\textbf{G}}}^{\top }$ using tools such as GCTA or PLINK [1, 13]. Calculation of the GRM requires $O(N^2M)$ time. However, as this calculation can be massively parallelized, it places little practical limitation on either *N* or *M*.

By giving standardized SNPs the same weight, the preceding definition of the GRM makes tacit assumptions about the relationship between allele frequency and linkage disequilibrium on the one hand and SNP effect sizes on the other. Other tools, such as LDAK [14], can be used to construct a GRM that assigns different weights to the SNPs, thereby incorporating different assumptions about SNP effect sizes. Importantly, MGREML can use any valid GRM in binary format as input, irrespective of its precise definition and irrespective of whether it is calculated using PLINK, GCTA, or LDAK.

The parameters of interest in the univariate model are $\sigma _{G_{tt}}$ and $\sigma _{E_{tt}}$, where $\sigma _{G_{tt}}$ denotes the additive genetic variance of Trait *t* captured by the available SNPs and $\sigma _{E_{tt}}$ the remaining variance in Trait *t*. The latter quantity is sometimes referred to as the environmental variance, even though this name can be somewhat misguiding, since $\sigma _{E_{tt}}$ simply reflects all variance in Trait *t* that is not tagged by the additive linear effects of the available SNPs and covariates [6]. In spite of the subtleties in its definition, we stick to the convention of calling this term the environmental variance.

In this model, $h^2_{\text{SNP}}$ of Trait *t* can be defined as $h^2_{\text{SNP}}(t) = \sigma _{G_{tt}} \left( \sigma _{G_{tt}} + \sigma _{E_{tt}} \right) ^{-1}$. In essence, univariate GREML quantifies the degree to which genetic similarity between individuals, as tagged by the SNPs used to construct the GRM, maps to trait similarity.

Notice here that REML does not estimate $\varvec{\beta }_t$ directly. Instead, REML controls for the fixed-effect covariates by considering so-called error contrasts [15, 16]. More specifically, REML estimation is equivalent to maximum-likelihood estimation applied to ${\textbf{K}}_t{\textbf{y}}_t$, where the rows of matrix ${\textbf{K}}_t$ form a basis of the left null space of ${\textbf{X}}_t$. However, once REML estimates of $\sigma _{G_{tt}}$ and $\sigma _{E_{tt}}$ are obtained, one can readily calculate the generalized least squares estimator of the fixed effects $\varvec{\beta }_t$ [1, 9]. This option is implemented in both GCTA and MGREML.

The univariate LMM can be generalized to a multivariate LMM [17, 18], which can be used to jointly estimate genetic covariance and environmental covariance between Traits $t=1,\ldots ,T$ and $s=1,\ldots ,T$, denoted by $\sigma _{G_{ts}}$ and $\sigma _{E_{ts}}$ respectively. Using the same notation as seen in the original derivations of MGREML [9], this multivariate LMM can be written as follows:

$$\begin{aligned} \left( \begin{array}{c} {\textbf{y}}_1 \\ \vdots \\ {\textbf{y}}_T \end{array} \right) \sim {\mathcal {N}} \left( \left( \begin{array}{ccc} {\textbf{X}}_1 &{} &{} {\textbf{0}} \\ &{} \ddots &{} \\ {\textbf{0}} &{} &{} {\textbf{X}}_T \end{array} \right) \left( \begin{array}{c} \varvec{\beta }_1 \\ \vdots \\ \varvec{\beta }_T \end{array} \right) , {\textbf{V}}_G \otimes {\textbf{A}} + {\textbf{V}}_E \otimes {\textbf{I}}_{N} \right) , \end{aligned}$$

where ‘$\otimes$’ denotes the Kronecker product. In this model, ${\textbf{V}}_G$ is the $T \times T$ genetic variance matrix and ${\textbf{V}}_E$ the $T \times T$ environmental variance matrix. Now, the genetic correlation between Traits *t* and *s* is defined as $\rho _{G} (t,s) = \sigma _{G_{ts}} \left( \sigma _{G_{tt}} \sigma _{G_{ss}} \right) ^{-0.5}$ [2], where $\sigma _{G_{ts}}$ is element *t*, *s* from ${\textbf{V}}_G$.

### Computational complexity

The variance matrix of the multivariate model (i.e., ${\textbf{V}}_G \otimes {\textbf{A}} + {\textbf{V}}_E \otimes {\textbf{I}}_{N}$) is dense, rendering naïve REML estimation infeasible for large *N* and *T*, as mere evaluation of the log-likelihood function already requires $O(N^3T^3)$ time. However, the time complexity can be drastically reduced by transforming the data using the eigenvalue decomposition (EVD) of the GRM [4, 9].

Let ${\textbf{Q}} \varvec{\Phi }{{\textbf{Q}}}^{\top }$ denote the EVD of ${\textbf{A}}$. Here, ${\textbf{Q}}$ denotes the matrix of eigenvectors and $\varvec{\Phi }$ the diagonal matrix of eigenvalues. MGREML defines matrix ${\textbf{P}}$ as the $n = N-L$ columns from ${\textbf{Q}}$ that correspond to the eigenvalues that are not among the *L* largest, and ${\textbf{D}}$ as the diagonal matrix with corresponding eigenvalues, $d_1, \ldots , d_n$.

Using this matrix ${\textbf{P}}$, MGREML transforms the data, and then reorders it such that (*i*) the variance matrix is block diagonal, enabling significant computational improvements, and (*ii*) the contribution of the *L* leading principal components from the genetic data to the variance matrix are eliminated, thus, correcting for population stratification [19] without introducing any additional fixed-effect covariates [9]. By default $L=20$, causing MGREML to control for even quite subtle population stratification. Users can specify a different value for *L* using the ––adjust-pcs option.

More specifically, the following model holds for $Tn \times 1$ vector ${\textbf{y}} = \textrm{vec} ( {{\textbf{Y}}}^{\top } {\textbf{P}})$ (where vec() denotes the vectorization operator):

$$\begin{aligned} {\textbf{y}} \sim {\mathcal {N}} \left( {\textbf{Z}} \varvec{\beta }, {\textbf{V}} \right) \textrm{ with \, } {\textbf{V}} = {\textbf{D}} \otimes {\textbf{V}}_G + {\textbf{I}}_{n} \otimes {\textbf{V}}_E, \end{aligned}$$

where ${\textbf{Z}} = ({{\textbf{Z}}}^{\top }_1 \; \cdots \; {{\textbf{Z}}}^{\top }_{n})^{\top }$, ${\textbf{Z}}_j = \left( {\textbf{I}}_T \otimes {{\textbf{x}}}^{\top }_j \right) {{\textbf{S}}}^{\top }$, ${{\textbf{x}}}^{\top }_j$ is $1 \times k$ row *j* from ${{\textbf{P}}}^{\top } {\textbf{X}}$, and

$$\begin{aligned} {\textbf{S}} = \left( \begin{array}{ccc} {\textbf{S}}_1 &{} &{} {\textbf{0}} \\ &{} \ddots &{} \\ {\textbf{0}} &{} &{} {\textbf{S}}_T \end{array} \right) . \end{aligned}$$

Omitting the constant, the corresponding log-likelihood function is given by

$$\begin{aligned} \ell \left( {\textbf{y}}, \varvec{\theta }\right) = -\frac{1}{2}\left( \textrm{log} \left| {\textbf{V}} \right| + \textrm{log} \left| {\textbf{Z}}^{\top } {\textbf{V}}^{-1} {\textbf{Z}} \right| + {{\textbf{y}}}^{\top } {\textbf{M}} {\textbf{y}} \right) , \end{aligned}$$

where ${\textbf{M}} = {\textbf{V}}^{-1} - {\textbf{V}}^{-1} {\textbf{Z}} \left( {\textbf{Z}}^{\top } {\textbf{V}}^{-1} {\textbf{Z}} \right) ^{-1} {\textbf{Z}}^{\top } {\textbf{V}}^{-1}$ [1]. This log-likelihood function depends on $\textrm{log} \left| {\textbf{V}} \right|$ and quadratic forms of the type $q = {{\textbf{w}}}^{\top } {\textbf{V}}^{-1} {\textbf{w}}$. Importantly, ${\textbf{V}}$ is a highly sparse, block-diagonal matrix, where diagonal block *j* equals ${\textbf{V}}_j = d_j {\textbf{V}}_G + {\textbf{V}}_E$, with ${\textbf{V}}_G$ and ${\textbf{V}}_E$ being functions of the parameter vector $\varvec{\theta }$.

As a result of this block-diagonal structure, these quadratic forms and log-determinants can be written as a sum of *n* independent contributions, where each contribution comes from a $T \times T$ block. MGREML can calculate the contribution of any given block in $O(T^2)$ time. Concordantly, the log-likelihood function can be evaluated in $O(NT^2)$ time. Similarly, the gradient (i.e., the vector of partial derivatives of the log-likelihood function with respect to $\varvec{\theta }$) can also be calculated in $O(NT^2)$ time.

MGREML retains its computational efficiency in case there are a limited number of fixed effects covariates. However, if the number of covariates grows large, MGREML will get slower. Nevertheless, as MGREML controls for population stratification without having to introduce any fixed effects for that purpose, a limited number of fixed-effect covariates suffices in a typical empirical application.

The average information (AI) algorithm, a variation on Newton’s method [1, 20], is ill-suited for MGREML estimation for large *T*, since that algorithm involves repeated calculation of the AI matrix, which requires $O(NT^4)$ time per iteration for a saturated model [9]. Specifically, a saturated model has $T(T-1)$ free parameters. Thus, the AI matrix has $T(T-1) \times T(T-1)$ elements, where each element involves a calculation requiring *O*(*N*) time, placing overall complexity at $O(NT^4)$.

To avoid having to calculate the AI matrix in every iteration, MGREML instead uses a Broyden–Fletcher–Goldfarb–Shanno (BFGS) algorithm [21] in combination with a golden-section line search to estimate $\varvec{\theta }$. Importantly, a BFGS iteration has roughly the same computational complexity as a gradient-descent iteration yet a higher rate of convergence across iterations. Thus, a BFGS algorithm balances computational complexity per iteration and rate of convergence across iterations.

The BFGS algorithm is initialized such that the first iteration is equivalent to a gradient-descent iteration with golden-section search. Evaluations of the log-likelihood function and its gradient suffice for application of the golden-section search and BFGS algorithm, putting the overall time complexity of MGREML at $O(NT^2)$ per iteration.

For large *T*, the BFGS algorithm can exhibit unstable behavior, in which case relevant quantities are reinitialized such that first next BFGS iteration is again equivalent to a gradient-descent iteration with golden-section search. If instability persists, MGREML switches to the AI algorithm for a single iteration. In our experience, such expensive ‘interventions’ are needed only sparingly and are effective in resolving numerical instabilities in MGREML estimation.

Once MGREML has converged, the variance matrix of ${\widehat{\varvec{\theta }}}$ is estimated using the AI matrix [20]. In addition, a delta method is used to obtain the standard error (SE) of $h^2_{\text{SNP}}$ and $\rho _G$ estimates. Although calculation of the AI matrix, as indicated, is expensive, this calculation only needs to be carried out once. Moreover, MGREML users can specify the ––no-se option to forgo calculation of the AI matrix and SEs altogether after convergence of the BFGS algorithm.

### Factor structures

**Fig. 1:**
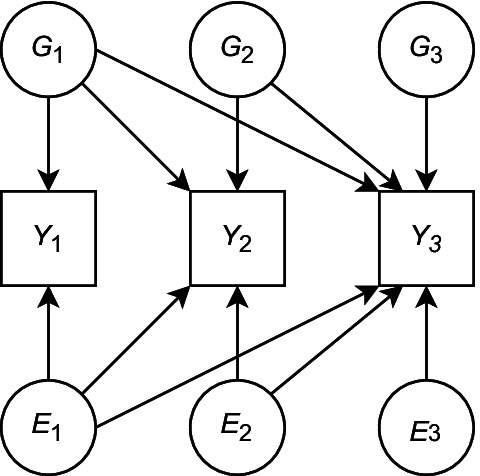
A saturated genetic and environmental factor model for three traits

By default, MGREML assumes a saturated model for both ${\textbf{V}}_G$ and ${\textbf{V}}_E$. An example of such a saturated model for $T=3$ traits is shown in Fig. 1. Letting $\gamma _{tf}$ (resp. $\varepsilon _{tf}$) denote the effect of genetic (environmental) Factor *f* on Trait *t*, the saturated model for $T=3$ traits can be written as follows:

$$\begin{aligned} {\textbf{V}}_G&= {\textbf{C}}_G {{\textbf{C}}}^{\top }_G \text{ and } {\textbf{V}}_E = {\textbf{C}}_E {{\textbf{C}}}^{\top }_E, \text{ where } \\ {\textbf{C}}_G&= \left( \begin{array}{ccc} \gamma _{11} &{} 0 &{} 0 \\ \gamma _{21} &{} \gamma _{22} &{} 0 \\ \gamma _{31} &{} \gamma _{32} &{} \gamma _{33} \end{array} \right) \text{ and } {\textbf{C}}_E = \left( \begin{array}{ccc} \varepsilon _{11} &{} 0 &{} 0 \\ \varepsilon _{21} &{} \varepsilon _{22} &{} 0 \\ \varepsilon _{31} &{} \varepsilon _{32} &{} \varepsilon _{33} \end{array} \right) . \end{aligned}$$

For *T* traits in general, a saturated model for ${\textbf{V}}_G$ (resp. ${\textbf{V}}_E$) can be described in terms of a lower triangular matrix of free genetic (environmental) coefficients ${\textbf{C}}_G$ (${\textbf{C}}_E$) where ${\textbf{V}}_G = {\textbf{C}}_G {{\textbf{C}}}^{\top }_G$ (${\textbf{V}}_E = {\textbf{C}}_E {{\textbf{C}}}^{\top }_E$).

Here, we generalize this approach by allowing ${\textbf{C}}_G$ (resp. ${\textbf{C}}_E$) to be a $T \times F_G$ ($T\times F_E$) matrix of which a pre-defined subset of the $TF_G$ ($TF_E$) elements are free, while the other elements are constrained to zero, reflecting an arbitrary factor model with $F_G$ ($F_E$) genetic (environmental) factors. Both factor models need to satisfy standard identification requirements in structural equation modeling [22]. Under this framework, the implied genetic (resp. environmental) variance matrix ${\textbf{V}}_G$ (${\textbf{V}}_E$) is always at least positive (semi)-definite. In other words, provided the user-specified model is identified, MGREML always yields valid correlation matrices.

MGREML users can specify a main model, comprising a genetic factor model and an environmental factor model. In case a user also specifies a nested model, MGREML performs a classical likelihood-ratio test (LRT) [23], to infer whether the fit of the main factor model is significantly better than that of the nested model.

In total, users can specify at most four factor models: (*1A*) the main genetic factor model, (*1B*) the main environmental factor model, (*2A*) the nested genetic factor model, and (*2B*) the nested environmental factor model. For example, a user can specify a main genetic factor model where there is only one genetic factor for all traits and a nested genetic factor model, where the traits have no genetic variance at all (i.e., there is no genetic factor), while the environmental factor model is saturated both in the main model as well as the nested model.

A factor model specification for MGREML is effectively a binary $T \times F$ matrix stored as a plain text file, where *F* denotes the number of factors. More specifically, in a given model, for $f=1,\ldots ,F$ and $t=1,\ldots ,T$, if Factor *f* has a free path coefficient to Trait *t*, element *t*, *f* of the binary matrix equals one and otherwise that element equals zero.

Let $C_{G_A}$ (resp. $C_{E_A}$) denote the number of free coefficients in the main genetic (environmental) factor model and let $C_{G_0}$ (resp. $C_{E_0}$) be defined analogously for the nested model. Finally, let $\ell _{A}$ (resp. $\ell _{0}$) denote the log-likelihood of the main (nested) model. Now, the LRT statistic is calculated by MGREML as $\text{LRT} = 2(\ell _{A}-\ell _{0})$, which under standard maximum likelihood estimation (MLE) assumptions [24] and nestedness of the models is $\chi ^2\left( (C_{G_A} + C_{E_A}) - (C_{G_0} + C_{E_0}) \right)$ distributed.

**Table 1:** Specification of a genetic factor model for height and body mass index (BMI) observed at five different points in time (denoted by subscripts indicating waves 7, 8, ..., 11)

Trait	*G*$_{height }$	*G*$_{BMI }$	*G*$_{shared }$
*height*$_{7}$	1	0	1
*height*$_{8}$	1	0	1
*height*$_{9}$	1	0	1
*height*$_{10}$	1	0	1
*height*$_{11}$	1	0	1
*BMI*$_{7}$	0	1	1
*BMI*$_{8}$	0	1	1
*BMI*$_{9}$	0	1	1
*BMI*$_{10}$	0	1	1
*BMI*$_{11}$	0	1	1

**Fig. 2:**
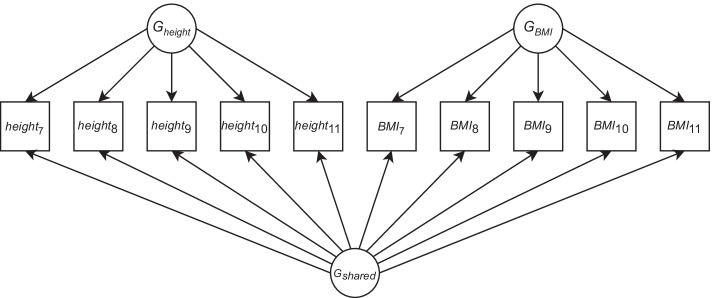
A genetic factor model for height and body mass index (BMI) observed at five different points in time (denoted by subscripts indicating waves 7, 8, ..., 11)

An example of a genetic factor model that MGREML users can specify is shown in Table 1. The corresponding structural equation model for ${\textbf{V}}_G$ which MGREML fits under that specification is shown in Fig. 2. The environmental factors shaping ${\textbf{V}}_E$ are not shown here, for clarity of the figure. We use this genetic factor model in our empirical application. In this example, the first genetic factor captures the genetic signal shared between all height measurements, the second genetic factor captures the genetic signal shared between all measurements of body mass index (BMI), and the third factor captures the genetic overlap between height and BMI (i.e., the genetic correlation).

## Results

### Simulation study

To test the validity of MGREML estimates of genetic correlations and underlying factor structures, we generated 100 independent datasets with $N=20,000$ individuals and $T=10$ traits with SNP-based $h^2 = 50\%$. In Simulation 1, we set $\rho _G$ to the same value across all combinations of traits. In Simulation 2, we simulate two clusters of five traits by setting $\rho _G$ to random values within clusters and to zero between clusters. In Simulation 3, we consider one additional dataset with $N=20,000$ individuals and $T=50$ traits with SNP-based $h^2 = 50\%$ and $\rho _G=0$. The simulation design is fully described in the Supplementary Information [see Additional File 1].

As MGREML estimation is a specific form of MLE, we expect MGREML to yield consistent estimates of the population parameters, provided standard MLE assumptions hold [24]. That is, as *N* increases, each parameter estimate converges to the true value. The results of Simulation 1 support the claim that MGREML yields consistent estimates of $h^2_{\text{SNP}}$ and $\rho _G$ across the full range of feasible values for $\rho _G$ [see Additional File 1: Tables S1–S4]. The SEs of estimates also align with the standard deviations of estimates across the generated datasets. Estimates have lower SEs when interdependence across traits is higher (i.e., higher $\vert \rho _G \vert$).

**Fig. 3:**
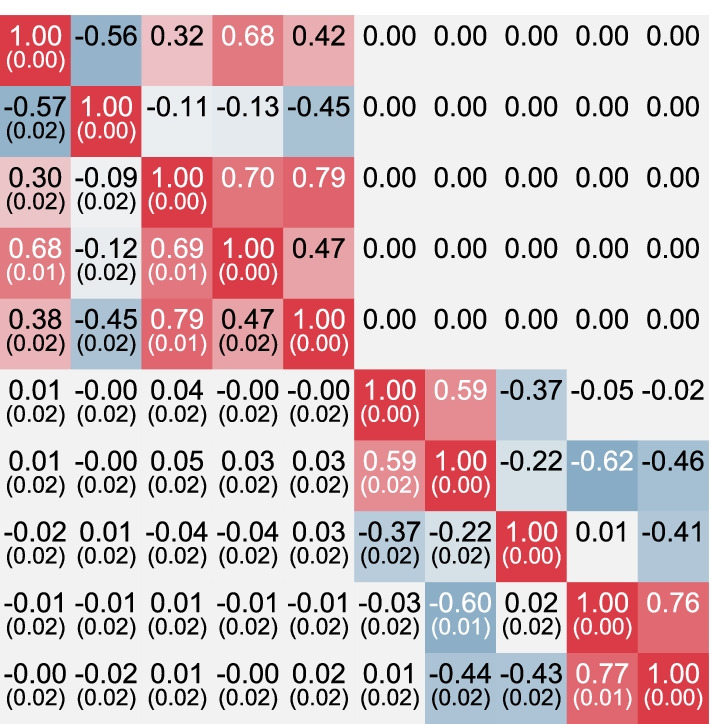
Typical MGREML estimate of a genetic correlation ($\rho _G$) matrix in Simulation 2. True genetic correlations ($\rho _G$’s) are shown above the diagonal. Estimated $\rho _G$’s (standard error between parentheses) are shown below the diagonal

The results of Simulation 2 show that MGREML also yields consistent estimates when the degrees of freedom in the model is larger than necessary [see Additional File 1: Table S5]. Estimates closely reflect the implied factor structure, as illustrated in Fig. 3 which shows MGREML estimation results for the first dataset. When comparing the fit of the appropriate factor model and the saturated model using an LRT, we find that resulting *p*-values closely follow the correct theoretical distribution [see Additional File 1: Figure S1].

The results of Simulation 3 show that MGREML can readily estimate and compare factor models for $T=50$ traits observed in $N=20,000$ individuals, involving 50 fixed effects and including calculation of SEs, on a single notebook with two 2.7 GHz cores and 16 GB of RAM in less than one hour. In addition, on more powerful machines, MGREML estimation can handle at least up until $T=200$ traits and $N=20,000$ individuals [9].

### Empirical application

To illustrate the ability of MGREML to estimate a factor model and test whether it fits the data better than a nested model, we use data on $N=6,425$ unrelated individuals from the US Health and Retirement Study (HRS) [25], for whom we analyze repeated measures of height and BMI in five consecutive waves of data collection (Waves 7–11). The HRS is a longitudinal panel study that surveys a representative sample of approximately 20,000 individuals aged 51 years and older (and their spouses) in the United States. Further details (e.g., quality control filters and descriptive statistics) are provided in the Supplementary Information [see Additional File 1].

As a baseline model, we start by assuming height and BMI both have no genetic variance (Model I). Given that previous $h^2_{\text{SNP}}$ estimates for height and BMI are considerably greater than zero [26, 27] (e.g., ${\widehat{h}}^2_{\text{SNP}} (\text{height}) = 43\%$ with $\text{SE} = 2\%$ and ${\widehat{h}}^2_{\text{SNP}} (\text{BMI}) = 21\%$ with $\text{SE} = 2\%$ [28]), we also consider an alternative model with one genetic factor for the height observations and one genetic factor for the BMI observations (Model II), which corresponds to the first two columns of the factor model shown in Table 1 labeled *G*$_{height }$ and *G*$_{BMI }$.

Although we expect Model II to have a far better fit than Model I, Model II still assumes there is no genetic correlation between height and BMI. Yet, there is ample evidence that height and BMI are genetically correlated traits [9, 29] (e.g., ${\widehat{\rho }}_G ( \text{height},\text{BMI}) = -0.14$ with $\text{SE} = 0.04$ [9]). Therefore, we also consider a third model in which we introduce a shared genetic factor that affects both the height and BMI observations (Model III), accounting for the genetic overlap between these two traits. Model III corresponds to the factor model shown in Table 1 (where the shared factor is labeled *G*$_{shared }$) and equivalently in Fig. 2. In all three models, we assume a saturated environmental factor model.

With the HRS surveying a representative sample of individuals aged 51 years and older (and their spouses), it seems unlikely that the unique and the shared genetic architecture of height and BMI will drastically change for individuals in our analysis sample between the biennial waves of data collection. Therefore, we *a priori* believe Model III to be most suitable for the data. That is, we expect this to be the most parsimonious model that is able to capture both the unique and the shared genetic component of height and BMI across waves. At the same time, taking aforementioned estimates of $h^2_{\text{SNP}}$ and $\rho _G$ for height and BMI at face value, and using the online GCTA-GREML power calculator [30], we find that the statistical power to detect $\rho _G (\text{height},\text{BMI}) \ne 0$ in this sample is only 21.8%. Hence, Model II might not be rejected in favor of Model III simply due to lack of statistical power. Details of this power calculation are described in the Supplementary Information [see Additional File 1].

In the application to data on repeated measures of height and BMI, we first compare the fit of Model I and Model II. We find that Model II, as expected, fits the data better than Model I (LRT=72.03, degrees of freedom=10, *p*-value=$1.79 \times 10^{-11}$). Thus, the null model of no genetic variance is rejected in favor of a model in which (1) height has genetic variance, (2) BMI has genetic variance, yet (3) height and BMI have no genetic correlation. When we compare the fit of Model II and Model III, we do not find an improvement in fit (LRT=11.11, degrees of freedom=10, *p*-value=0.349). Thus, the model without genetic correlation between height and BMI is not rejected in favor of a model with genetic overlap, in line with our power calculation.

## Conclusion

Accurate estimates of genetic correlations and genetic factor structures across multiple traits help to understand their shared etiology and aid in finding likely causal relationships [29, 31]. As such, estimation and inference based on genetic and environmental factor models may contribute to the design of future genetic and functional studies.

Here, we derived a statistical framework (1) to model and estimate such factor models using individual-level SNP data and (2) to test hypotheses regarding these factor models. Using simulations and an empirical application, we confirmed the validity of this statistical framework.

This framework is implemented in our freely available command-line tool MGREML, which has simple input options for this purpose. MGREML accepts user-specified genetic and environmental factor models as input, and performs estimation and inference based thereon. Even on a single machine, this tool can readily be applied to data on 20,000 individuals and 50 traits.

## Supplementary Information

**Additional file 1.** Supplementary Information.

## Data Availability

*Mandated data deposition* All data used in this manuscript are freely available via public-access repositories. Specifically, we obtained access to the genetic data of the Health and Retirement Study (HRS) through the Database of Genotypes and Phenotypes (dbGaP): https://www.ncbi.nlm.nih.gov/gap/ (dbGaP application 3544). The RAND HRS data, produced by the RAND Center for the Study of Aging, containing the phenotype data, can be accessed via the HRS website: https://hrsdata.isr.umich.edu/data-products/rand/. Researchers who wish to link genotype and phenotype data from the HRS must apply for access via the HRS website: https://hrsdata.isr.umich.edu/data-products/genetic-cross-reference/. *Software and code* The MGREML tool and the code for the simulation study are freely available via the GitHub repository for this project: · **Project name:** MGREML · **Project home page (including tutorial):**https://github.com/devlaming/mgreml/ · **Operating system:** platform independent · **Programming language:** Python 3.x. · **Other requirements:** Python packages networkx, numpy, pandas, psutil, scipy, and tqdm · **License:** GNU GPL v3 · **Any restrictions to use by non-academics:** as stipulated by GNU GPL v3

## Abbreviations

AI: Average information
BFGS algorithm: Broyden–Fletcher–Goldfarb–Shanno algorithm
BMI: Body mass index
EVD: Eigenvalue decomposition
GCTA: Genome-wide complex trait analysis
GREML: Genomic-relatedness-based restricted maximum likelihood
GRM: Genomic-relatedness matrix
GWAS: Genome-wide association study
HRS: Health and Retirement Study
LMM: Linear mixed model
LRT: Likelihood-ratio test
MGREML: Multivariate genomic-relatedness-based restricted maximum likelihood
MLE: Maximum likelihood estimation
RAM: Random-access memory
REML: Restricted maximum likelihood
SE: Standard error
SNP: Single-nucleotide polymorphism

## References

[1] Yang J, Lee SH, Goddard ME, Visscher PM (2011). GCTA: a tool for genome-wide complex trait analysis. Am J Hum Genet.

[2] Lee SH, Yang J, Goddard ME, Visscher PM, Wray NR (2012). Estimation of pleiotropy between complex diseases using single-nucleotide polymorphism-derived genomic relationships and restricted maximum likelihood. Bioinformatics..

[3] Benjamin DJ, Cesarini D, Chabris CF, Glaeser EL, Laibson DI, Gudnason V (2012). The promises and pitfalls of genoeconomics. Annu Rev Econ..

[4] Lee SH, Van der Werf JHJ (2016). MTG2: an efficient algorithm for multivariate linear mixed model analysis based on genomic information. Bioinformatics..

[5] Evans LM, Tahmasbi R, Vrieze SI, Abecasis GR, Das S, Gazal S (2018). Comparison of methods that use whole genome data to estimate the heritability and genetic architecture of complex traits. Nat Genet..

[6] Lee JJ, Chow CC (2014). Conditions for the validity of SNP-based heritability estimation. Hum Genet..

[7] Witte JS, Visscher PM, Wray NR (2014). The contribution of genetic variants to disease depends on the ruler. Nat Rev Genet..

[8] Yang J, Zeng J, Goddard ME, Wray NR, Visscher PM (2017). Concepts, estimation and interpretation of SNP-based heritability. Nat Genet..

[9] De Vlaming R, Slob EAW, Jansen PR, Dagher A, Koellinger PD, Groenen PJF (2021). Multivariate analysis reveals shared genetic architecture of brain morphology and human behavior. Commun Biol..

[10] Schumacker RE, Lomax RG (2016). A beginner’s guide to structural equation modeling.

[11] Grotzinger AD, Rhemtulla M, de Vlaming R, Ritchie SJ, Mallard TT, Hill WD (2019). Genomic structural equation modelling provides insights into the multivariate genetic architecture of complex traits. Nat Hum Behav..

[12] Patterson HD, Thompson R (1971). Recovery of inter-block information when block sizes are unequal. Biometrika..

[13] Chang CC, Chow CC, Tellier LC, Vattikuti S, Purcell SM, Lee JJ (2015). Second-generation PLINK: rising to the challenge of larger and richer datasets. GigaScience..

[14] Speed D, Hemani G, Johnson MR, Balding DJ (2012). Improved heritability estimation from genome-wide SNPs. Am J Hum Genet..

[15] Harville DA (1974). Bayesian inference for variance components using only error contrasts. Biometrika..

[16] Casella G, Searle SR. On a matrix identity useful in variance component estimation. Biometrics Unit Technical Reports. 1985; p. BU-875-M.

[17] Meyer K (1985). Maximum likelihood estimation of variance components for a multivariate mixed model with equal design matrices. Biometrics..

[18] Lynch M, Walsh B (1998). Genetics and analysis of quantitative traits.

[19] Price AL, Patterson NJ, Plenge RM, Weinblatt ME, Shadick NA, Reich D (2006). Principal components analysis corrects for stratification in genome-wide association studies. Nat Genet..

[20] Gilmour AR, Thompson R, Cullis BR (1995). Average information REML: an efficient algorithm for variance parameter estimation in linear mixed models. Biometrics..

[21] Nocedal J, Wright SJ (2006). Numerical optimization.

[22] Bollen KA (1989). Structural equations with latent variables.

[23] Wilks SS (1938). The large-sample distribution of the likelihood ratio for testing composite hypotheses. Ann Math Stat..

[24] Newey WK, McFadden D. Chapter 36: Large sample estimation and hypothesis testing. vol. 4 of Handbook of Econometrics. Elsevier; 1994. p. 2111–2245.

[25] Sonnega A, Faul JD, Ofstedal MB, Langa KM, Phillips JWR, Weir DR (2014). Cohort profile: the Health and Retirement Study (HRS). Int J Epidemiol..

[26] Yang J, Benyamin B, McEvoy BP, Gordon S, Henders AK, Nyholt DR (2010). Common SNPs explain a large proportion of the heritability for human height. Nat Genet..

[27] Yang J, Manolio TA, Pasquale LR, Boerwinkle E, Caporaso N, Cunningham JM (2011). Genome partitioning of genetic variation for complex traits using common SNPs. Nat Genet..

[28] De Vlaming R, Okbay A, Rietveld CA, Johannesson M, Magnusson PK, Uitterlinden AG (2017). Meta-GWAS Accuracy and Power (MetaGAP) calculator shows that hiding heritability is partially due to imperfect genetic correlations across studies. PLoS Genet..

[29] Bulik-Sullivan B, Finucane HK, Anttila V, Gusev A, Day FR, Loh PR (2015). An atlas of genetic correlations across human diseases and traits. Nat Genet..

[30] Visscher PM, Hemani G, Vinkhuyzen AA, Chen GB, Lee SH, Wray NR (2014). Statistical power to detect genetic (co) variance of complex traits using SNP data in unrelated samples. PLoS Genet..

[31] O’Connor LJ, Price AL (2018). Distinguishing genetic correlation from causation across 52 diseases and complex traits. Nat Genet..

