# Supplementary material for: Multivariate estimation of factor structures of complex traits using SNP-based genomic relationships
Source: BMC Bioinformatics. 2022 Jul 27;23:305. doi: 10.1186/s12859-022-04835-3 (PMC9327374; doi:10.1186/s12859-022-04835-3)
Supplement: Supplementary file 1 — Additional file 1. Supplementary Information. [file 12859_2022_4835_MOESM1_ESM.pdf]

Additional File 1

Supplementary Information for “Multivariate Estimation of Factor Structures of Complex Traits Using SNP-based Genomic Relationships”

Ronald de Vlaming, Eric A.W. Slob, Patrick J.F. Groenen, and Cornelius A. Rietveld

1. Simulations

Simulating genotype and phenotype data

We simulate R independent datasets (*runs*). In each run, we generate data for M = 20,000 single-nucleotide polymorphisms (SNPs) with minor allele frequency ≥ 5%, for a sample comprising N = 20,000 unrelated individuals. In each run, we compute the genomic-relatedness matrix (GRM) for MGREML estimation [1]. We then generate T traits, each with the same SNP-based heritability (h^2 = 50%). These traits have pre-specified true genetic correlations (ρ_G), which differ across the simulation settings. The genetic correlation of trait t and s is here defined as the correlation of the additive linear genetic component for trait t and s, respectively (e.g., see [2]).

The code for the simulation study is available at `https://github.com/devlaming/mgreml/` in subdirectory `simulations`. Here, `run1.sh` contains the bash code to perform one run of the simulation study and `simulatedata.py` contains Python code to simulate data that is called upon by `run1.sh`. The general structure of the code is as follows:

1. Simulate genetic data for N = 20,000 unrelated individuals on M = 20,000 SNPs:
   a. For SNP $m = 1, \ldots, M$:
      1) Draw allele frequency $f_m \sim \text{Beta}(0.35, 0.35)$ and repeat until $f_m \in [0.05, 0.95]$.
      2) For individual $j = 1, \ldots, N$: draw genotype $G_{jm} \sim \text{Binom}(2, f_m)$.
      3) Compute empirical allele frequency: $m_j = (G_{1m} + \ldots + G_{Nm})[2N]^{-1}$.
      4) For individual $j = 1, \ldots, N$: standardize genotype: $X_{jm} = (G_{jm} - 2m_j)[2m_j(1 - m_j)]^{-0.5}$.
   b. Let $\mathbf{X}$ denote the resulting $N \times M$ matrix of standardized genotypes.
   c. Compute $\mathbf{A} = M^{-1}\mathbf{XX}^\top$ and store this GRM in binary format.
2. Simulate genetic factors and environmental effects, with no covariance between factors, conditional on the genotypes:
   a. For genetic factor $f = 1, \ldots, F_G$:
      1) Draw SNP effects: $\boldsymbol{\beta}_f \sim \text{N}(\mathbf{0}, \mathbf{I}_M)$.
      2) Compute genetic factor: $\mathbf{g}_f^* = \mathbf{X}\boldsymbol{\beta}_f$.
      3) Standardize $\mathbf{g}_f^*$ to have empirical mean zero and unit variance → $\mathbf{g}_f$.
   b. Let $\mathbf{G}$ denote the resulting $N \times F_G$ matrix of genetic factors.
   c. For environmental effects for trait $t = 1, \ldots, T$:
      1) Draw environmental effects: $\boldsymbol{\varepsilon}_t^* \sim \text{N}(\mathbf{0}, \mathbf{I}_N)$.
      2) Standardize $\boldsymbol{\varepsilon}_t^*$ to have empirical mean zero and unit variance → $\boldsymbol{\varepsilon}_t$.
   d. Let $\mathbf{E}$ denote resulting the $N \times T$ matrix of environmental effects.
3. Simulate T traits: $\mathbf{Y} = \surd(h^2)\mathbf{GW}_G + \surd(1 - h^2)\mathbf{E}$, where $\mathbf{W}_G$ is $F_G \times T$ matrix of genetic factor weights and where $h^2 = 0.50$.
4. Use the binary GRM and simulated traits to perform MGREML estimation.

Given the initial genetic factor weight matrix, denoted by $\mathbf{W}_G$*, we rescale each of the T columns to have unit length, yielding $\mathbf{W}_G$. We have three main simulation settings. In Simulation Setting 1 and 2 we consider T = 10 traits and R = 100 runs, while in Simulation

Setting 3 we consider $T = 50$ traits and $R = 1$ run. Across runs, we simulate different genetic datasets. Therefore, results for a given setting are completely independent across runs. In addition, within a given run, we draw new $\boldsymbol{\beta}_f$ and $\boldsymbol{\varepsilon}_f$ for each simulation setting and for each distinct value of ρ_G considered in Simulation Setting 1, creating considerable independence across settings and across values of ρ_G within a given run.

Simulation Setting 1

We fix the true ρ_G across traits to the same value. We consider four values of ρ_G in total:

1. $\rho_G = -(T-1)^{-1} = -0.111\ldots$ (i.e., the lowest possible fixed value for $T = 10$ traits).
   a. Define $\mathbf{R}_G = \rho_G \boldsymbol{\iota\iota}^\top + (1 - \rho_G)\mathbf{I}_T$.
   b. Find the eigenvalue decomposition of $\mathbf{R}_G$ denoted by $\mathbf{B\Lambda B}^\top$.
   c. Set $\mathbf{W}_G{}^* = \boldsymbol{\Lambda}^{0.5}\mathbf{B}^\top$.
2. $\rho_G = 0.00$ (i.e., no genetic correlation across traits). Here, the expression for the phenotypes reduces to $\mathbf{Y} = \sqrt{(0.5)}\mathbf{G} + \sqrt{(0.5)}\mathbf{E}$.
3. $\rho_G = 0.50$. For details on how to construct $\mathbf{R}_G$ and $\mathbf{W}_G{}^*$ see Step 1 of this simulation setting, replacing $\rho_G = -(T-1)^{-1}$ by $\rho_G = 0.50$.
4. $\rho_G = 1.00$. Here, the expression for the phenotypes reduces to $\mathbf{Y} = \sqrt{(0.50)}\mathbf{g}\boldsymbol{\iota}^\top + \sqrt{(0.50)}\mathbf{E}$, where $\mathbf{g}$ is the $N \times 1$ vector for the single genetic factor needed in this model.

For the data simulated here, we estimate a fully saturated model in each run. Supplementary Tables S1–S4 show the resulting h^2 estimates, ρ_G estimates, their standard errors (SEs), averaged across runs, and the standard deviation in ρ_G estimates across runs, for the four distinct values of ρ_G.

We observe that MGREML provides consistent estimates of the true genetic correlation structure, and that the estimates become more precise (i.e., have lower SEs) when interdependence across traits increases (i.e., higher $|\rho_G|$). SEs are in line with standard deviations, validating the delta method implemented in MGREML. In the range of setups considered here, only for $\rho_G = 1.00$ SEs are considerably larger than standard deviations. This finding means MGREML is slightly conservative in terms of SEs in extreme scenarios.

Simulation Setting 2

We divide the traits into two clusters of five traits, with random ρ_G within clusters and $\rho_G = 0.00$ between traits in different clusters. Again, for each trait $h^2 = 50\%$. A typical run is shown in Figure 1 in the main text. We use the following steps to find an appropriate $\mathbf{W}_G{}^*$:

1. Initialize $\mathbf{W}_G{}^*$ as 10×10 matrix of i.i.d. draws from $N(0,1)$.
2. Set the elements in 5×5 lower-left block of $\mathbf{W}_G{}^*$ to zero.
3. Set the elements in 5×5 upper-right block of $\mathbf{W}_G{}^*$ to zero.

For the data simulated here, for each run we estimate both a nested model (first set of five genetic factors affecting Traits 1–5, second set of five genetic factors affecting Traits 6–10, and no environmental correlation) and a fully saturated model. Supplementary Table S5 shows the root-mean-square error (RMSE) calculated from the estimation error in ρ_G across all runs.

We observe that MGREML provides accurate estimates of the true genetic-correlation structure, even when the degrees of freedom in the model is larger than necessary (i.e., when a fully saturated model is estimated, while the data-generating process is not fully saturated). With respect to the ρ_G estimates that can be non-zero under the nested model, RMSE of those estimates is only marginally smaller than RMSE of the corresponding ρ_G estimates under the fully saturated model.

Supplementary Table S1. Average estimates over 100 simulation runs when $\rho_G = -1/9$ and $h^2 = 50\%$ for all generated traits. The h^2 estimates (with standard error between parentheses) are shown in the first column; The ρ_G estimates (with standard error between parentheses) are shown below the diagonal in the subsequent columns; The standard deviation of the ρ_G estimates across simulations runs is shown above the diagonal.

	h^2	Trait 1	Trait 2	Trait 3	Trait 4	Trait 5	Trait 6	Trait 7	Trait 8	Trait 9	Trait 10
Trait 1	0.499 (0.010)		[0.019]	[0.015]	[0.016]	[0.019]	[0.019]	[0.016]	[0.019]	[0.017]	[0.016]
Trait 2	0.499 (0.010)	−0.109 (0.018)		[0.017]	[0.019]	[0.018]	[0.018]	[0.019]	[0.018]	[0.019]	[0.018]
Trait 3	0.501 (0.010)	−0.112 (0.018)	−0.112 (0.018)		[0.018]	[0.019]	[0.017]	[0.017]	[0.017]	[0.019]	[0.020]
Trait 4	0.499 (0.010)	−0.111 (0.018)	−0.112 (0.018)	−0.110 (0.018)		[0.019]	[0.017]	[0.017]	[0.019]	[0.016]	[0.016]
Trait 5	0.499 (0.010)	−0.110 (0.018)	−0.112 (0.018)	−0.112 (0.018)	−0.110 (0.018)		[0.019]	[0.016]	[0.018]	[0.019]	[0.017]
Trait 6	0.499 (0.010)	−0.112 (0.018)	−0.109 (0.018)	−0.115 (0.018)	−0.109 (0.018)	−0.112 (0.018)		[0.017]	[0.017]	[0.017]	[0.017]
Trait 7	0.499 (0.010)	−0.113 (0.018)	−0.109 (0.018)	−0.110 (0.018)	−0.111 (0.018)	−0.108 (0.018)	−0.109 (0.018)		[0.015]	[0.018]	[0.019]
Trait 8	0.499 (0.010)	−0.110 (0.018)	−0.110 (0.018)	−0.112 (0.018)	−0.109 (0.018)	−0.109 (0.018)	−0.113 (0.018)	−0.114 (0.018)		[0.019]	[0.019]
Trait 9	0.500 (0.010)	−0.109 (0.018)	−0.110 (0.018)	−0.107 (0.018)	−0.111 (0.018)	−0.111 (0.018)	−0.110 (0.018)	−0.111 (0.018)	−0.112 (0.018)		[0.018]
Trait 10	0.499 (0.010)	−0.108 (0.018)	−0.110 (0.018)	−0.111 (0.018)	−0.111 (0.018)	−0.108 (0.018)	−0.114 (0.018)	−0.111 (0.018)	−0.110 (0.018)	−0.110 (0.018)	

Supplementary Table S2. Average estimates over 100 simulation runs when $\rho_G = 0$ and $h^2 = 50\%$ for all generated traits. The h^2 estimates (with standard error between parentheses) are shown in the first column; The ρ_G estimates (with standard error between parentheses) are shown below the diagonal in the subsequent columns; The standard deviation of the ρ_G estimates across simulations runs is shown above the diagonal.

	h^2	Trait 1	Trait 2	Trait 3	Trait 4	Trait 5	Trait 6	Trait 7	Trait 8	Trait 9	Trait 10
Trait 1	0.502 (0.010)		[0.019]	[0.019]	[0.016]	[0.017]	[0.020]	[0.020]	[0.018]	[0.019]	[0.020]
Trait 2	0.500 (0.010)	−0.002 (0.019)		[0.016]	[0.017]	[0.019]	[0.019]	[0.018]	[0.018]	[0.019]	[0.019]
Trait 3	0.499 (0.010)	−0.002 (0.019)	0.001 (0.019)		[0.018]	[0.022]	[0.018]	[0.021]	[0.019]	[0.018]	[0.018]
Trait 4	0.502 (0.010)	0.004 (0.018)	−0.001 (0.019)	0.001 (0.019)		[0.018]	[0.019]	[0.018]	[0.017]	[0.018]	[0.018]
Trait 5	0.500 (0.010)	0.001 (0.019)	0.001 (0.019)	0.001 (0.019)	0.002 (0.019)		[0.018]	[0.018]	[0.017]	[0.018]	[0.017]
Trait 6	0.499 (0.010)	−0.001 (0.019)	−0.001 (0.019)	0.000 (0.019)	0.000 (0.019)	0.004 (0.019)		[0.017]	[0.016]	[0.018]	[0.016]
Trait 7	0.501 (0.010)	−0.001 (0.019)	0.000 (0.019)	0.002 (0.019)	0.002 (0.019)	−0.002 (0.019)	−0.004 (0.019)		[0.018]	[0.019]	[0.020]
Trait 8	0.500 (0.010)	0.001 (0.019)	−0.003 (0.019)	−0.001 (0.019)	0.000 (0.019)	−0.002 (0.019)	0.002 (0.019)	0.002 (0.019)		[0.018]	[0.018]
Trait 9	0.500 (0.010)	0.001 (0.019)	−0.001 (0.019)	0.002 (0.019)	0.001 (0.019)	0.003 (0.019)	0.000 (0.019)	0.002 (0.019)	0.001 (0.019)		[0.020]
Trait 10	0.499 (0.010)	−0.003 (0.019)	−0.001 (0.019)	0.000 (0.019)	0.002 (0.019)	0.002 (0.019)	0.000 (0.019)	0.000 (0.019)	0.001 (0.019)	−0.001 (0.019)	

Supplementary Table S3. Average estimates over 100 simulation runs when $\rho_G = 0.5$ and $h^2 = 50\%$ for all generated traits. The h^2 estimates (with standard error between parentheses) are shown in the first column; The ρ_G estimates (with standard error between parentheses) are shown below the diagonal in the subsequent columns; The standard deviation of the ρ_G estimates across simulations runs is shown above the diagonal.

	h^2	Trait 1	Trait 2	Trait 3	Trait 4	Trait 5	Trait 6	Trait 7	Trait 8	Trait 9	Trait 10
Trait 1	0.499 (0.009)		[0.016]	[0.017]	[0.014]	[0.015]	[0.018]	[0.015]	[0.015]	[0.015]	[0.016]
Trait 2	0.500 (0.009)	0.501 (0.016)		[0.016]	[0.015]	[0.013]	[0.017]	[0.015]	[0.016]	[0.015]	[0.015]
Trait 3	0.500 (0.009)	0.499 (0.016)	0.500 (0.016)		[0.017]	[0.016]	[0.015]	[0.015]	[0.015]	[0.017]	[0.017]
Trait 4	0.499 (0.009)	0.501 (0.016)	0.502 (0.016)	0.498 (0.016)		[0.016]	[0.017]	[0.017]	[0.016]	[0.016]	[0.017]
Trait 5	0.499 (0.009)	0.503 (0.016)	0.501 (0.016)	0.499 (0.016)	0.500 (0.016)		[0.014]	[0.016]	[0.015]	[0.016]	[0.018]
Trait 6	0.500 (0.009)	0.499 (0.016)	0.501 (0.016)	0.497 (0.016)	0.501 (0.016)	0.498 (0.016)		[0.015]	[0.016]	[0.015]	[0.017]
Trait 7	0.502 (0.009)	0.499 (0.016)	0.498 (0.016)	0.500 (0.016)	0.498 (0.016)	0.501 (0.016)	0.500 (0.016)		[0.014]	[0.015]	[0.015]
Trait 8	0.502 (0.009)	0.502 (0.016)	0.498 (0.016)	0.499 (0.016)	0.497 (0.016)	0.501 (0.016)	0.499 (0.016)	0.500 (0.016)		[0.013]	[0.014]
Trait 9	0.500 (0.009)	0.501 (0.016)	0.502 (0.016)	0.500 (0.016)	0.499 (0.016)	0.500 (0.016)	0.502 (0.016)	0.503 (0.016)	0.499 (0.016)		[0.015]
Trait 10	0.499 (0.009)	0.502 (0.016)	0.501 (0.016)	0.500 (0.016)	0.500 (0.016)	0.503 (0.016)	0.501 (0.016)	0.498 (0.016)	0.500 (0.016)	0.501 (0.016)	

Supplementary Table S4. Average estimates over 100 simulation runs when $\rho_G = 1$ and $h^2 = 50\%$ for all generated traits. The h^2 estimates (with standard error between parentheses) are shown in the first column; The ρ_G estimates (with standard error between parentheses) are shown below the diagonal in the subsequent columns; The standard deviation of the ρ_G estimates across simulations runs is shown above the diagonal.

	h^2	Trait 1	Trait 2	Trait 3	Trait 4	Trait 5	Trait 6	Trait 7	Trait 8	Trait 9	Trait 10
Trait 1	0.505 (0.008)		[0.006]	[0.006]	[0.005]	[0.005]	[0.006]	[0.005]	[0.006]	[0.006]	[0.006]
Trait 2	0.504 (0.008)	0.991 (0.010)		[0.006]	[0.006]	[0.006]	[0.006]	[0.006]	[0.006]	[0.005]	[0.005]
Trait 3	0.504 (0.008)	0.990 (0.010)	0.991 (0.010)		[0.005]	[0.005]	[0.005]	[0.006]	[0.006]	[0.005]	[0.006]
Trait 4	0.505 (0.008)	0.991 (0.010)	0.991 (0.010)	0.992 (0.010)		[0.005]	[0.005]	[0.006]	[0.005]	[0.006]	[0.005]
Trait 5	0.504 (0.008)	0.991 (0.010)	0.991 (0.010)	0.991 (0.010)	0.991 (0.010)		[0.006]	[0.006]	[0.006]	[0.006]	[0.006]
Trait 6	0.503 (0.008)	0.991 (0.010)	0.990 (0.010)	0.991 (0.010)	0.991 (0.010)	0.991 (0.010)		[0.005]	[0.005]	[0.006]	[0.005]
Trait 7	0.506 (0.008)	0.991 (0.010)	0.991 (0.010)	0.991 (0.010)	0.991 (0.010)	0.991 (0.010)	0.991 (0.010)		[0.005]	[0.006]	[0.006]
Trait 8	0.504 (0.008)	0.990 (0.010)	0.991 (0.010)	0.991 (0.010)	0.991 (0.010)	0.991 (0.010)	0.991 (0.010)	0.991 (0.010)		[0.006]	[0.006]
Trait 9	0.504 (0.008)	0.991 (0.010)	0.990 (0.010)	0.991 (0.010)	0.990 (0.010)	0.991 (0.010)	0.990 (0.010)	0.991 (0.010)	0.991 (0.010)		[0.005]
Trait 10	0.505 (0.008)	0.991 (0.010)	0.991 (0.010)	0.990 (0.010)	0.991 (0.010)	0.991 (0.010)	0.990 (0.010)	0.991 (0.010)	0.990 (0.010)	0.991 (0.010)	

Supplementary Table S5. Root-mean-square error (RMSE) calculated from the estimation errors in the ρ_G estimates across simulation runs in Simulation 2 (two clusters of five traits, with random ρ_G within clusters and ρ_G = 0.00 between clusters). RMSE for estimates from the fully saturated model are shown below the diagonal; RMSE for estimates from the nested model are shown above the diagonal.

	Trait 1	Trait 2	Trait 3	Trait 4	Trait 5	Trait 6	Trait 7	Trait 8	Trait 9	Trait 10
Trait 1		0.014	0.013	0.012	0.014					
Trait 2	0.016		0.014	0.015	0.013					
Trait 3	0.016	0.016		0.014	0.014					
Trait 4	0.015	0.017	0.016		0.013					
Trait 5	0.017	0.015	0.016	0.016						
Trait 6	0.019	0.016	0.018	0.018	0.018		0.013	0.014	0.014	0.013
Trait 7	0.020	0.019	0.018	0.017	0.022	0.016		0.013	0.012	0.014
Trait 8	0.019	0.018	0.019	0.017	0.020	0.016	0.014		0.013	0.013
Trait 9	0.019	0.017	0.021	0.017	0.020	0.015	0.015	0.015		0.015
Trait 10	0.017	0.017	0.020	0.020	0.019	0.017	0.016	0.016	0.017	

Supplementary Figure S1. Quantile-quantile (QQ) plots of the (a) $-\log_{10}$(p-values) and the (b) LRT statistics across simulation runs in Simulation Setting 2. The p-values are based on the likelihood-ratio test (LRT) assuming a χ^2 distribution with 70 degrees of freedom.

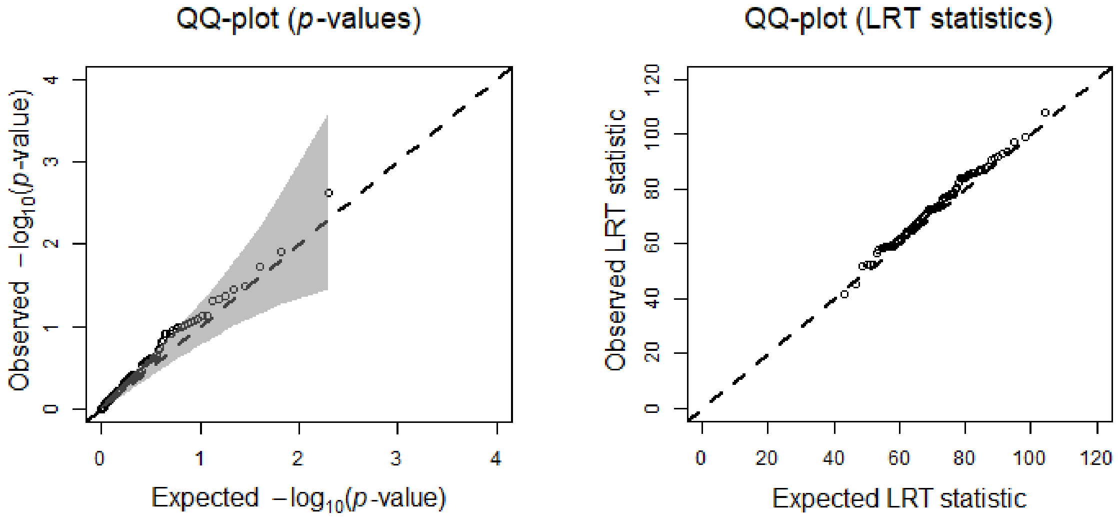

(a) (b)

Supplementary Figure S1 shows the quantile-quantile (QQ) plots of the MGREML likelihood-ratio test (LRT) statistics and corresponding $-\log_{10}$(p-values) across runs, when comparing the fit of the nested model to the fit of the fully saturated model in each run. Given the data-generating process, the LRT statistics should follow a χ^2 distribution with 70 degrees of freedom. This expectation is confirmed by the QQ plots, because no appreciable deviation from the 45-degree line is observed.

Simulation Setting 3

To investigate runtime of MGREML when comparing factor models, we consider one additional simulation run in accordance with Simulation Setting 1, with ρ_G = 0.00, while considering T = 50 traits instead of ten traits. For this simulated data, we estimate a saturated main model and a nested model assuming one idiosyncratic genetic factor for each trait (i.e., a

nested model in accordance with the true value $\rho_G = 0.00$), and compare the fit of these two models using a likelihood-ratio test. In both models, we include an intercept as a fixed-effect covariate that applies to each trait. Thus, there are 50 fixed effects in total in both models. We find that MGREML finishes this analysis in approximately 42 minutes on a single notebook with two 2.7 GHz cores and 16 GB of RAM.

2. Application

To illustrate the ability of MGREML to distinguish between realistic and unrealistic factor models, we employ data from the US Health and Retirements Study (HRS). The HRS is a longitudinal panel study that surveys a representative sample of approximately 20,000 individuals aged 51 years and older (and their spouses) in the United States of America [3].

To construct the GRM, we use the 2012 release of genetic data. The genetic data in this release were obtained from the DNA samples collected from HRS participants in the years between 2006 and 2008. Genotyping was carried out using the Illumina Human Omni-2.5 Quad BeadChip. After imposing the quality control filters recommended by the genotyping center, removal of non-autosomal SNPs and SNPs with Minor Allele Frequency (MAF) < 0.05, genotyping call rate < 95% and Hardy-Weinberg p-value < 10^{-6}, and selection of unrelated individuals from European ancestry with < 5% SNP missingness, we construct the GRM for a sample of 8,652 individuals based on 1,188,298 SNPs.

Phenotypic data from the biennial waves of data collection in the HRS are harmonized by the RAND cooperation. We exploit information about human height (m) and body mass index (BMI, kg/m^2) as available in the RAND HRS Longitudinal File 2016 (V2). We selected phenotypic data from the five consecutive waves of data collection with the lowest number of missing values (Waves 7–11, years 2004–2012). We do not impose further quality control filters on the phenotype data.

Supplementary Table S6. Descriptive statistics of height, body mass index (BMI), sex, and birth year in the sample from the Health and Retirement Study (N = 6,570).

	Mean	Standard deviation	Minimum	Maximum
Height (Wave 7)	1.697	0.098	1.270	2.108
Height (Wave 8)	1.690	0.100	1.245	2.261
Height (Wave 9)	1.688	0.100	1.245	2.248
Height (Wave 10)	1.685	0.100	1.321	2.248
Height (Wave 11)	1.682	0.101	1.219	2.083
BMI (Wave 7)	27.485	5.274	13.700	58.400
BMI (Wave 8)	27.868	5.470	10.600	67.300
BMI (Wave 9)	27.925	5.568	14.500	64.600
BMI (Wave 10)	27.834	5.682	7.000	63.800
BMI (Wave 11)	27.689	5.759	13.100	62.600
Sex (1=Female; 0=Male)	0.588	0.492	0.000	1.000
Birth year	1938.976	9.767	1911.000	1974.000

There are 6,570 individuals with full information on the 10 traits (5 for height, 5 for BMI) in our model. In our analyses, we control for (standardized) birth year, birth year^2, birth year^3, sex, sex × birth year, sex × birth year^2, and sex × birth year^3. MGREML also adjusts for the first 20 principal components of the genomic-relatedness matrix to control for subtle forms of population stratification.

Supplementary Table S6 provides descriptive statistics of the HRS analysis sample. Due to the exclusion of individuals with a genetic relatedness larger than 0.025, the actual analysis sample comprises 6,425 individuals.

3. Power calculation to detect genetic correlation between height and BMI

We use the GCTA-GREML power calculator [4] to compute the statistical power to reject the null of no genetic correlation between height and BMI by applying MGREML to the HRS data, using the following settings:

- Type of analysis = Bivariate;
- Type of traits = Two quantitative traits;
- Sample size Trait #1 (height) = Sample size Trait #2 (BMI) = 6,425 (i.e., the sample size after MGREML has applied the relatedness cut-off);
- Phenotypic correlation = 0.053 (based on the correlation between average height across Waves 7 – 11 and average BMI across Waves 7 – 11);
- Genetic correlation = −0.14 (based on [1]);
- Heritability Trait #1 (height) = 0.43 (based on [5]);
- Heritability Trait #2 (BMI) = 0.21 (based on [5]);
- Are the same samples used for both traits = Yes;
- Type I error rate = 0.05 (default value); and
- Variance of the SNP-derived genetic relationships = 2×10^{-5} (default value).

The resulting probability that the null (i.e., no genetic correlation) is correctly rejected is 21.8%. Conversely, the probability of a Type II error is 78.2%.

References

1. De Vlaming R, Slob EAW, Jansen PR, Dagher A, Koellinger PD, Groenen PJF, et al. Multivariate analysis reveals shared genetic architecture of brain morphology and human behavior. Commun. Biol. 2021;4:1180.
2. Lynch M, Walsh B. Genetics and analysis of quantitative traits. 1st ed. Sunderland, MA: Sinauer; 1998.
3. Sonnega A, Faul JD, Ofstedal MB, Langa KM, Phillips JWR, Weir DR. Cohort profile: the Health and Retirement Study (HRS). Int. J. Epidemiol. 2014;43:576-85.
4. Visscher PM, Hemani G, Vinkhuyzen AA, Chen GB, Lee SH, Wray NR, et al. Statistical power to detect genetic (co)variance of complex traits using SNP data in unrelated samples. PLoS Genet. 2014;10:e1004269.
5. De Vlaming R, Okbay A, Rietveld CA, Johannesson M, Magnusson PK, Uitterlinden AG, et al. Meta-GWAS Accuracy and Power (MetaGAP) calculator shows that hiding heritability is partially due to imperfect genetic correlations across studies. PLoS Genet. 2017;13:e1006495.
